# Expanding the V1-MT model to the estimation of perceived fluid direction

**DOI:** 10.1038/s41598-025-99069-7

**Published:** 2025-04-26

**Authors:** Takahiro Kawabe

**Affiliations:** NTT Communication Science Laboratories, 3-1, Morinosato Wakamiya, Atsugi, Kanagawa 243-0198 Japan

**Keywords:** Motion, Liquid flow direction, V1-MT model, Weighted average, Human behaviour, Neural encoding

## Abstract

Humans can readily perceive the direction of liquid flow, yet computational modeling of this process remains challenging due to the complexity of non-rigid motion. Previous models based on neural activities in the primary visual cortex (V1) and the middle temporal area (MT) have been effective in explaining rigid motion perception. In this study, we extend the V1-MT model to address the perception of liquid flow direction. Participants observed video clips of liquid flow and reported the perceived direction, while the V1-MT model was used to predict these perceptions. The winner-take-all approach failed to accurately capture the observed perceptions. In contrast, a weighted mean of directional energies yielded strong predictions, highlighting that the human visual system spatially integrates directional energies from non-rigid motion components. These findings broaden the applicability of the V1-MT model to non-rigid motion and provide insights into how the visual system bridges the gap between computational models of rigid and non-rigid motion perception.

## Introduction

Human observers can discriminate various material properties of liquids based on visual image features. For instance, the viscosity of liquids can be perceived from motion speeds^1^ and the shape of liquid outlines^2,3^, while the transparency of liquids is estimated from spatiotemporal deformation frequencies^4^ and the spatial coverage of linear image motion^5^. Despite these advances, a critical aspect—how humans perceive liquid flow direction—remains largely unexplored. The inherent complexity of predicting liquid flow direction from dynamic visual cues arises from the highly non-rigid nature of liquids. This study aims to address this gap by investigating the mechanisms underlying liquid flow direction perception, situating the findings within the broader framework of non-rigid motion perception in visual motion processing.

Understanding the perception of liquid flow direction requires reviewing the general principles of motion detection. Human vision detects motion through a sequential two-stage processing framework^6–8^. The first stage analyzes spatiotemporal luminance patterns, conceptualized as detecting spatiotemporally tilted orientation signals^9^. The second stage integrates outputs from units sensitive to adjacent spatiotemporal orientations across space, resolving the aperture problem^10^ and the determining of motion direction^7,8^. These stages are attributed to distinct cortical regions: V1 detects spatiotemporal energy^11,12^, while MT determines motion direction^13–15^.

While the two-stage process is effective for simple and naturalistic stimuli^16^, its applicability to liquid flow perception, characterized by non-rigid and complex motion, remains unexplored. Although the V1-MT model has been widely used for rigid motion stimuli, its ability to represent liquid flow direction is unclear.

Perceptual representation from MT neural activity is generally computed using two approaches: winner-take-all and vector averaging. The winner-take-all model assumes that perceptual decisions are driven by the most active neurons, while the vector averaging model integrates signals across a population of neurons, weighted by their activity levels^17^. Traditionally, the winner-take-all model has been widely used to infer perceived motion direction from MT activity^18^ while the vector averaging model has been shown to predict both motion direction judgments^19,20^ and patterns of saccadic and pursuit eye movements^21^. Psychophysical studies further support that motion signals are spatially integrated through vector averaging for two-dimensional (i.e., pattern) motion^22,23^.

Understanding which computational strategy underlies liquid flow direction perception is critical for extending motion perception models to complex, non-rigid stimuli. The present study compares the winner-take-all and vector averaging models to determine which better explains the perception of liquid flow direction. This represents the first attempt to evaluate whether the two-stage V1-MT model, traditionally applied to rigid motion, can be adapted to complex, non-rigid liquid motions.

## Methods

### Psychophysical experiments

#### Participants

A total of 14 participants (4 females) and 16 participants (8 females) took part in Experiments 1 and 2, respectively. The mean ages (± SD) were 24.42 ± 7.11 years for Experiment 1 and 26.62 ± 8.42 years for Experiment 2. Participants were recruited through a human resource agency in Japan and received monetary compensation based on criteria determined by the agency, which were not disclosed to the researchers. All participants were naive to the specific purposes of the experiments. Ethical approval for the study was obtained from the Ethics Committee of NTT Communication Science Laboratories (approval number: R06-013). The experiments adhered to the principles of the 2013 Declaration of Helsinki. Written informed consent was obtained from all participants prior to their participation.

#### Apparatus

Stimuli were presented on an LCD monitor (Display++, Cambridge Research Systems Inc., USA). The monitor’s luminance output was linearly calibrated using a luminance meter (LS-150, Konica Minolta Inc., Japan), with a luminance range of 0–144 cd/m^2^. A Mac Pro computer (Apple Inc., USA) was used to control stimulus presentation and data collection. The experimental scripts were written by using PsychoPy^24^.

#### Stimuli

Fifteen liquid flow clips were sourced from the internet and cropped to a 512 × 512-pixel subregion. This subregion was then downsampled to 256 × 256 pixels and presented to participants as stimulus clips. The clips were converted to grayscale, and their root mean square (RMS) contrast was adjusted to 0.23. In Experiment 1, each stimulus clip had a duration of 4 s (120 video frames at 30 Hz) and was repeatedly presented until participants made their judgments. In Experiment 2, each stimulus clip was trimmed to a duration of 167 ms (5 video frames at 30 Hz). The start frame of each clip was randomly selected from 24 possible frames (ranging from frame 0 to frame 115, in steps of 5). The clip was repeatedly presented until participants made their judgments, with an inter-stimulus interval of 500 ms.

#### Procedure

Each participant was individually tested in a dimly lit experimental room. A chin and headrest were used to stabilize their visual field. Each trial began with the participant pressing the space key. Participants were instructed to carefully observe the stimulus clip and report the perceived direction of liquid flow. This was done by aligning the direction of a rotating arrow, displayed around the stimulus clip, with the perceived flow direction. In Experiment 1, each participant completed 60 trials in a session, comprising 15 unique liquid flow scenes with four replications each. Participants performed a total of three sessions, resulting in 180 trials. In Experiment 2, each participant completed 60 trials in a session, consisting of 15 unique liquid flow scenes with four start frames each. Participants performed a total of 18 sessions, resulting in 1080 trials.

### V1-MT model

**Fig. 1:**
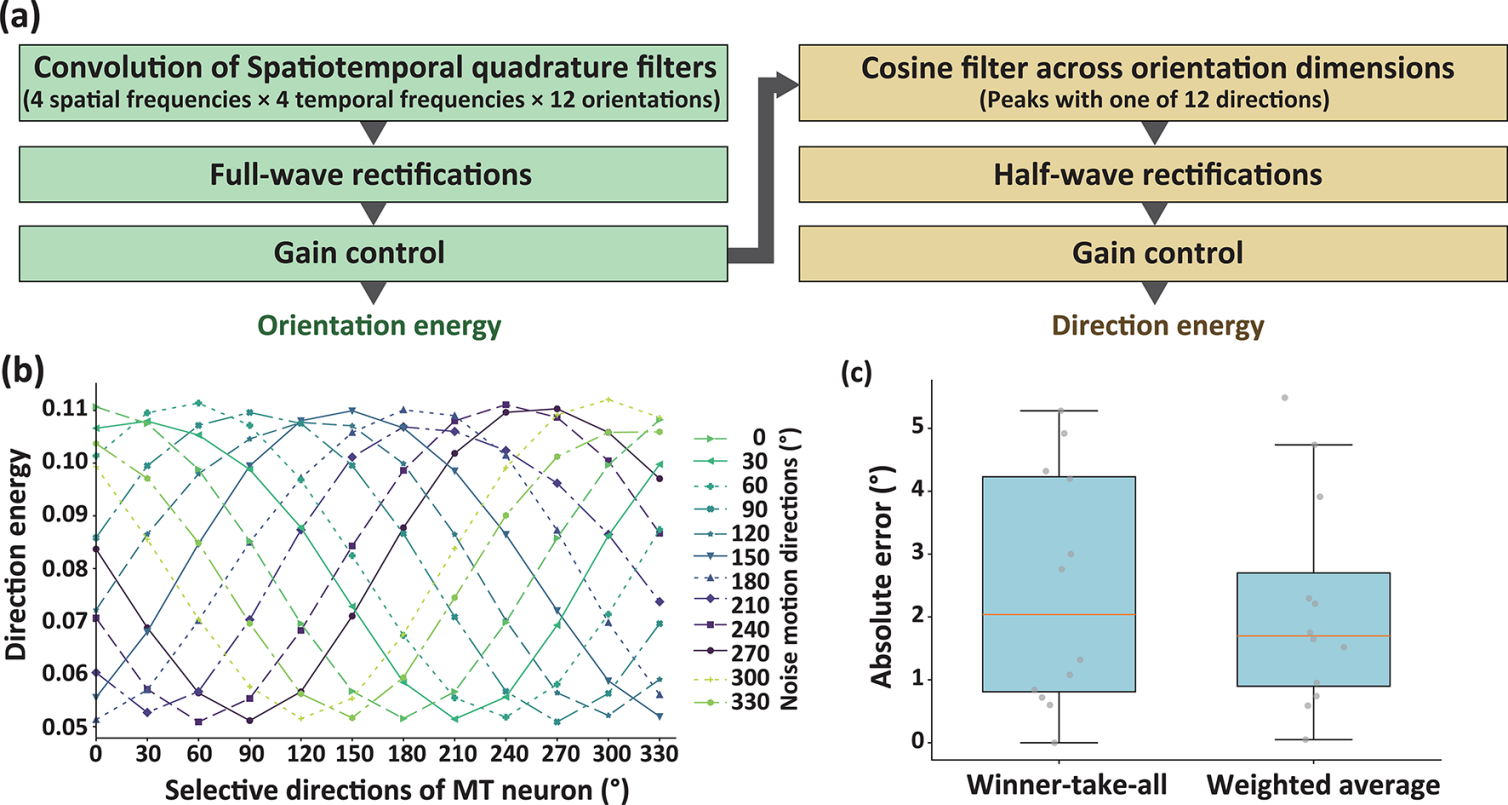
(**a**) The processing pipeline of the V1-MT model used in the present study, illustrating the stages from spatiotemporal quadrature filtering to the computation of direction energy. (**b**) The distribution of direction energy across selective directions of MT neurons for different noise motion directions. (**c**) A box plot comparing the absolute errors in motion direction estimation between the winner-take-all and weighted averaging models.

As shown in Fig. 1a, the model consisted of two processing stages: extracting orientation energy (which is often called “motion energy”) to simulate the properties of V1 and extracting direction energy to simulate the properties of MT. In the first stage, stimuli were convolved with three-dimensional kernels characterized by four spatial frequencies (0.4, 0.8, 1.6, and 3.2 cycles per degree), four temporal frequencies (0.75, 1.5, 3, and 6 Hz), and 12 orientations (0°, 30°, 60°, 90°, 120°, 150°, 180°, 210°, 240°, 270°, 300°, and 330°). Each kernel had a temporal length of 5 frames. The output of the convolution was full-wave rectified and gain-controlled, as described in a previous study. The resulting signals were considered motion energy. In the second stage, motion energy was integrated across nearby orientations using a cosine filter, as implemented in prior research^25^. The integrated signals were then half-wave rectified (i.e., ReLU applied) and gain-controlled to produce direction energy. In Experiment 1, a sequence consisting of 120 frames, each with a resolution of 256 × 256 pixels, representing liquid flow scenes, was analyzed as a whole. In Experiment 2, the same 120-frame sequence was divided into 24 subsets, each comprising 5 consecutive frames of 256 × 256 pixels, and these subsets were analyzed independently.

Two models were used to predict the perceived direction of liquid flow. In the winner-take-all model, dual von Mises functions were fitted to the direction energy distributions of each clip. Dual von Mises functions were used instead of single ones because some direction energy distributions exhibited dual peaks, making single functions inappropriate for accurate approximation. In fitting the function, we normalized direction energies by dividing the energies by their maximum values. The predicted direction was determined as the direction corresponding to the maximum value in the fitted function. The *r*^2^ for fitting was more than 0.95 for all stimulus clips.

In the weighted average model, the perceived direction was inferred by calculating the weighted average of the MT neuron activities using the following equation:

1 $$W=\text{arctan}(\frac{\sum {E}_{\theta }sin\theta }{\sum E}, \frac{\sum {E}_{\theta }cos\theta }{\sum E})$$

wherein *W* denotes the resultant predicted direction, *E* represents direction energies, and *θ* denotes the motion directions to which the MT neurons are tuned (ranging from 0° to 330° in steps of 30°).

## Results

### Evaluating basic performance of the V1-MT model

First, we evaluated the capability of the V1-MT model (Fig. 1a) to infer the motion direction of white noise stimuli. A two-dimensional white noise pattern (128 × 128 pixels, 15 frames) was presented moving in one of 12 directions (0° to 330° in 30° increments). Figure 1b illustrates the directional energy distributions for each motion direction, mapped to the direction selectivity of MT neurons. For each stimulus, the peak of directional energy was observed in MT neurons selective for the corresponding motion direction. To quantify the inferred direction, we fitted a single von Mises distribution to the directional energy distribution and identified the direction corresponding to the peak of the fitted function. This direction was considered the inferred direction under the winner-take-all model. The left box in Fig. 1c shows the distribution of absolute errors between the actual motion directions and the directions inferred by the winner-take-all model (median absolute error = 2.04°). Additionally, we calculated a weighted average of directional energies using Formula (1), as described in the Methods section. The right box in Fig. 1c presents the distribution of absolute errors between the actual motion directions and the directions inferred by the weighted average model (median absolute error = 1.70°). There was no statistical significance in the absolute errors between the two models (*t*(11) = 0.356, *p* = 0.72, *d* = 0.15). These findings indicate that both the winner-take-all and weighted averaging models can accurately infer the motion direction of rigid noise stimuli, with an error of approximately 2°.

### Application to the perceived direction of liquid flow

**Fig. 2:**
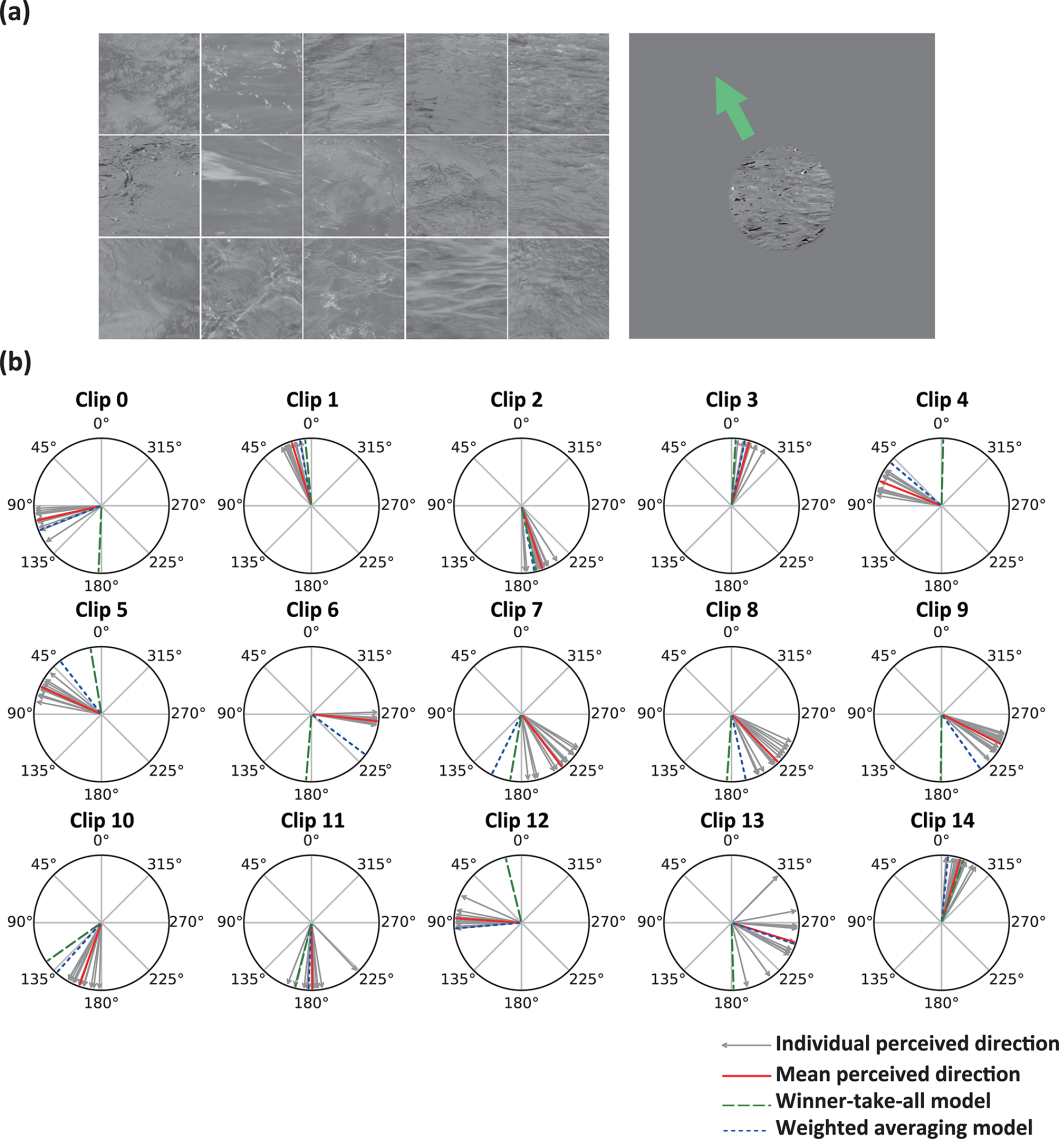
**(a**) Left: Snapshots of stimulus clips. Right: A snapshot of the experimental display showing a video frame of a stimulus clip accompanied by an arrow used by participants to report the perceived direction of liquid flow. (**b**) The red line represents the mean perceived direction for a stimulus clip across participants, while the gray thin arrows indicate individual perceived directions. The green dashed line and blue dotted line represent the directions inferred by the winner-take-all and weighted averaging models, respectively.

**Fig. 3:**
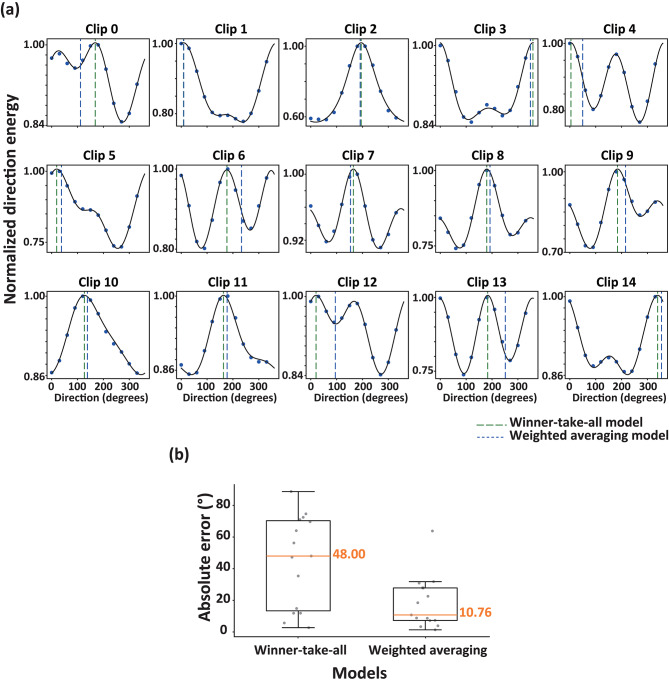
(**a**) Direction energies summed across MT neurons and fitted with a double von Mises function. The green dashed line and blue dotted line represent the directions inferred by the winner-take-all and weighted averaging models, respectively. (**b**) Box plots of the absolute errors between participants’ perceived direction and the inferred directions from the winner-take-all (left) and weighted averaging (right) models.

In Experiment 1, participants viewed a repeatedly presented 4-s clip of a liquid flow scene and reported the perceived direction of liquid flow by adjusting the direction of an arrow around the stimulus (Fig. 2a). Figure 2b shows the individual and mean perceived direction for all liquid flow scenes. We analyzed all clips using the V1-MT model and the results of the analysis are shown in Fig. 3a. To compare the winner-take-all and weighted averaging models (see “Methods” for details), we calculated the absolute errors for both models (Fig. 3b). The analysis revealed a significant difference between the two models (*t*(14) = 3.08, *p* = 0.0045, *d* = 1.13), with mean absolute errors of 48.00° for the winner-take-all model and 10.76° for the weighted averaging model. These results suggest that the perceived direction of liquid flow is more accurately captured by the weighted averaging model than by the winner-take-all model.

The advantage of employing a weighted averaging mechanism for determining the perceived direction of liquid flow in the visual system can be explained as follows. Liquid flow represents a non-rigid motion, and its motion vectors are spatiotemporally heterogeneous. In certain spatiotemporal points, signals corresponding to specific motion directions or velocities may transiently increase, rendering a winner-take-all approach, which relies solely on such signals, a risky strategy for determining motion direction. Instead, integrating motion vectors across a defined spatiotemporal window and calculating a weighted average provides a robust and plausible solution for perceiving liquid flow direction.

However, in Experiment 1, relatively long liquid flow clips were presented as stimuli, leaving it unclear whether the results reflect the spatial integration of motion vectors within the MT receptive field or whether they also involve temporally accumulated motion direction judgments.

**Fig. 4:**
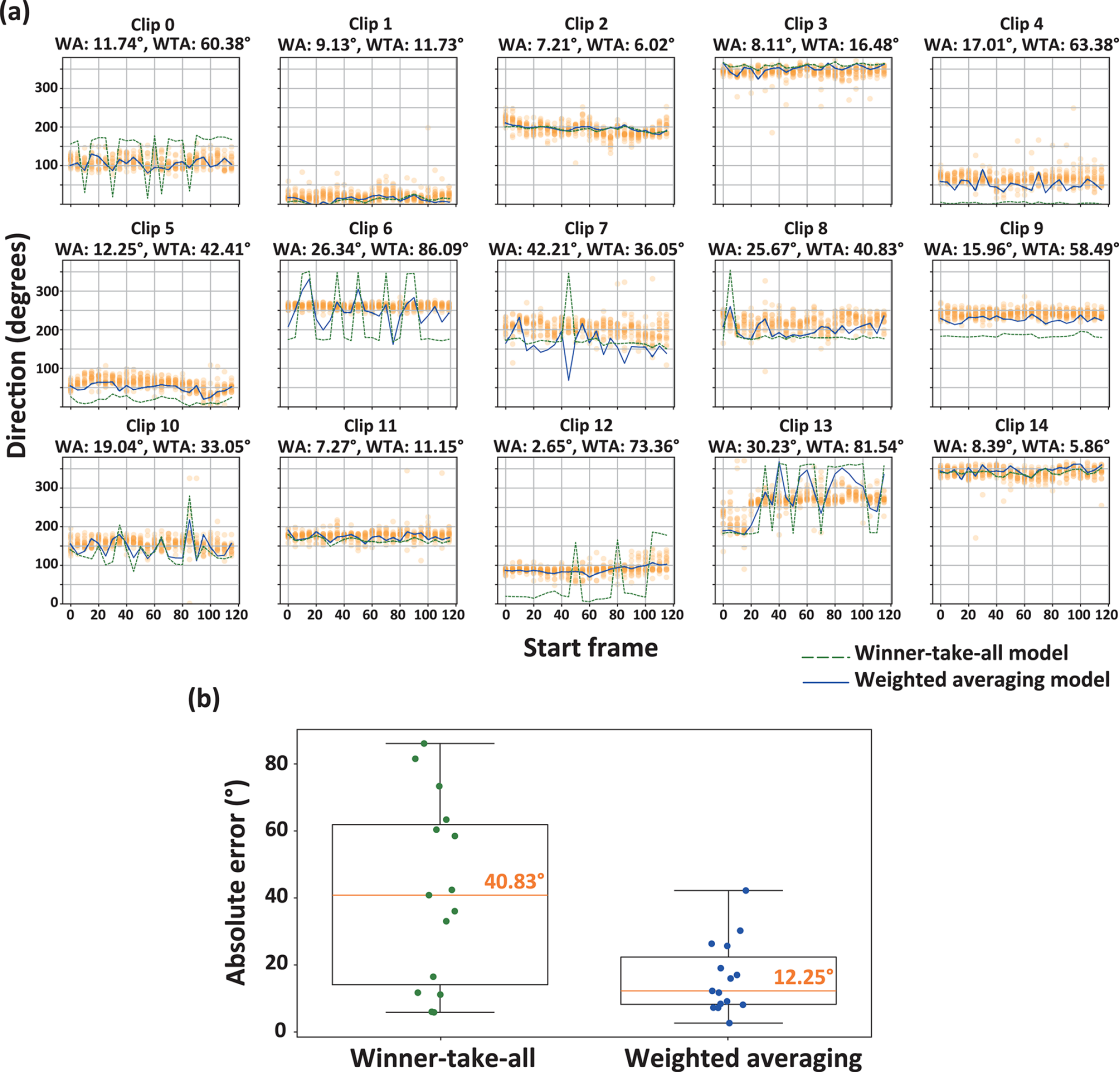
(**a**) Reported and inferred directions of liquid flow for each clip, plotted as a function of the start frame. Each clip consists of a 5-frame sequence of images. The values in the titles of the graphs indicate the absolute errors between the median reported direction and the inferred direction, as estimated by the weighted averaging (WA) and winner-take-all (WTA) models. (**b**) Box plots showing the absolute differences between reported and inferred directions for both the WA and WTA models across all clips.

Therefore, in Experiment 2, we tested whether the weighted averaging explanation still holds when the presentation duration is shortened from 4 to 0.167 s. Figure 4a shows the reported direction of liquid flow clips by 0.167 s and the inference of the direction based on both the weighted averaging and winter-take-all models. Median absolute errors were 12.25° for the weighted averaging model and 40.83° for the winner-take-all mechanism (Fig. 4b). We statistically compared the difference in the absolute errors between the two models and found significant difference, *t*(14) = 3.87, *p* = 0.002, *d* = 1.00. Moreover, there was no significant difference in the performance of the weighted averaging model between Experiments 1 and 2 (*t*(14) = 1.00, *p* = 0.29, *d* = 0.28).

The results demonstrated that the performance of the weighted averaging model was superior to that of the winner-take-all model, supporting the suggestion from Experiment 1 that the perceived direction of liquid flow is better inferred using a weighted averaging scheme than a winner-take-all scheme. Moreover, the performance of the weighted averaging model in Experiment 2 was comparable to its performance in Experiment 1, suggesting that the temporal accumulation of motion vectors may not play a substantial role in determining the perceived direction of liquid flow.

Although the presentation time was brief, the direction of liquid flow reported by the participants remained stable, which may be attributed to several factors. First, although the presentation duration in Experiment 2 was brief, the stimuli were presented repeatedly with a 500-ms inter-stimulus interval. This likely allowed participants to adjust their judgments about the flow direction until they were confident in their responses. Additionally, the liquid flow in the stimuli used in this study may have exhibited relatively stable flow directions, contributing to the consistency in participants’ reports. However, in Clip 13, the reported flow direction varied over time, which may be related to the large individual differences observed among participants in Experiment 1.

## Discussion

Our results demonstrated that the weighted averaging model contributes to the perception of liquid flow direction. Predictions based on the winner-take-all model significantly deviated from the perceived directions of liquid flow. As previously mentioned, this is likely due to the complexity of liquid flow, which makes relying on the output of a single neuron unsuitable for inferring its direction. Previous studies have shown that human visual processing employs a weighted averaging scheme when processing motion signals^17,19–21^. However, these studies have primarily focused on the applicability of this mechanism to the spatial integration of rigid motion. Our study suggests that weighted averaging may also be applicable to the spatial integration of non-rigid and complex motion, such as that observed in liquid flow.

It is important to note, however, that this model does not fully explain the perception of liquid flow direction. In both Experiments 1 and 2, the weighted averaging model successfully explained the perceived direction of liquid flow for approximately half of the clips. On the other hand, for the remaining half, the absolute error reached as high as 30°–60°, making it difficult to argue that the weighted averaging model provided an adequate explanation. Therefore, further investigation is essential to fully understand the perception of liquid flow direction.

One limitation of the present study is that, because the stimuli are natural video clips, it is not possible to determine the true direction of liquid flow within the scenes. As a result, we cannot distinguish whether the direction reported by the participants or the direction estimated by the model is more accurate. Accordingly, this study focuses on how well the existing motion model can account for the flow directions perceived and reported by human participants. To overcome this limitation, future research should employ stimuli generated from physically simulated liquid flows. This would allow for a comparison between the reported flow directions and the objectively known physical flow directions, enabling a more precise evaluation of perceptual accuracy.

It is important to discuss the strengths and limitations of the weighted averaging and winner-take-all (WTA) models in visual motion processing. As shown in Fig. 1, when inferring the motion direction of rigid noise motion, no significant difference was observed between the two models in terms of performance. These results suggest that for simple rigid translation, the WTA model is sufficient and computationally efficient. In contrast, when computing the flow direction of liquids—which involves more complex motion structures—the output of the WTA model becomes unreliable and potentially misleading. To accurately infer liquid flow direction, the visual system appears to adopt a weighted averaging strategy, incorporating signals from multiple motion detectors. This approach helps avoid the risk associated with relying solely on the most strongly activated detector.

Liquid flow is non-rigid and complex, and the visual information available for perception is equally intricate. Motion observed on the surface of the liquid does not always move orthogonally to the edge, as it does in rigid objects; some motion occurs parallel to the edge orientation. It is well known that motion perception is strongly influenced by orientation^26–29^ and shape^30,31^ information. We believe that motion illusions influenced by orientation and shape information, which are not readily captured by the V1-MT model, may play a role in the perception of liquid flow direction. Wavefronts that move orthogonally to their orientation axis on the image produce visual motion that is easily captured by the V1-MT model. However, wavefronts that move in a direction not orthogonal to their image orientation axis generate visual motion that is more difficult for the model to capture and are likely perceived with a bias influenced by the orientation axis.

This study did not address the non-rigidity of liquid flow, which is one of its complexities. Given that the V1-MT model is specialized for rigid motion, it is surprising that this model can even be applied to non-rigid motion like liquid flow. Since liquids lack a fixed shape, wavefronts that generate motion in a single direction do not persist for long. Such unstable wavefronts produce motion signals on the retinal image that are also unstable and prone to frequent changes. The direction energy distributions calculated in this study showed relatively unclear peaks, indicating that the motion signals detected by the V1-MT model are noisier compared to those from rigid motion.

Precisely because direction energy is noisy, the visual system’s behavior of determining the flow direction by weighted averaging of directional signals from the MT population is reasonable and warrants further investigation. While this study focused on liquid flow, it is likely that weighted averaging also plays a role in the perception of phenomena such as image distortions caused by heat refraction^32^ or the motion of smoke, which also generate non-rigid visual motion. Exploring the relationship between various natural phenomena and the behavior of the visual system could also provide intriguing insights into the evolutionary development of visual processing.

Finally, we would like to highlight the possibility that integrating the V1-MT model with other motion processing models, such as feature tracking^33^, could enhance the computational estimation of liquid flow directions. A recent study^34^ found that when two circular rings were firmly connected at an angle and rotated together at moderate speeds, observers perceived them as wobbling rather than rigidly linked. However, at slow speeds, they were perceived as rotating rigidly. The study aimed to computationally explain this perceptual transition from rigid to non-rigid appearance based on stimulus speed by integrating motion signals with salient shape cues. In their model, salient shape cues were captured using a feature tracking approach that incorporated Harris corner detectors and pattern motion-selective units. It is also possible that our stimuli contain varying degrees of turbulence across different spatial regions or between stimuli. However, our current model does not account for the influence of shape cues arising from turbulence in the perceptual determination of liquid flow directions. Future studies are needed to examine whether combining the motion energy model with feature tracking could improve the estimation accuracy of liquid flow directions.

## Supplementary Information

Supplementary Video 1.

## Data Availability

All data in this study are available upon request.
